# Cytoarchitecture and innervation of the mouse cochlear amplifier revealed by large‐scale volume electron microscopy

**DOI:** 10.1002/cne.25137

**Published:** 2021-03-18

**Authors:** Haoyu Wang, Shengxiong Wang, Yan Lu, Ying Chen, Wenqing Huang, Miaoxin Qiu, Hao Wu, Yunfeng Hua

**Affiliations:** ^1^ Department of Otolaryngology‐Head and Neck Surgery Shanghai Ninth People's Hospital Shanghai China; ^2^ Ear Institute, Shanghai Jiao Tong University School of Medicine Shanghai China; ^3^ Shanghai Key Laboratory of Translational Medicine on Ear and Nose Diseases Shanghai China; ^4^ Shanghai Institute of Precision Medicine Shanghai Ninth People's Hospital Shanghai China; ^5^ Putuo People's Hospital Tongji University Shanghai China

**Keywords:** mouse cochlea, outer hair cell, Deiter's cell, Type‐2 spiral ganglion neurons, medial olivocochlear efferent fibers, 3D electron microscopy

## Abstract

In mammalian cochlea, sound‐induced vibration is amplified by a three‐row lattice of Y‐shaped microstructures consisting of electromotile outer hair cell and supporting Deiters cell. This highly organized structure is thought to be essential for hearing of low‐level sounds. Prior studies reported differences in geometry and synaptic innervation of the outer hair cells between rows, but how these fine features are achieved at subcellular level still remains unclear. Using serial block‐face electron microscopy, we acquired few‐hundred‐micron‐sized cytoarchitecture of mouse organ of Corti at nanometer resolution. Structural quantifications were performed on the Y‐shapes as well as afferent and efferent projections to outer hair cells (OHCs). Several new features, which support the previously observed inter‐row heterogeneity, are described. Our result provides structural bases for the gradient of mechanical properties and diverse centrifugal regulation of OHC rows.

## Graphical abstract

By means of serial block‐face electron microscopy, Wang et al. revealed subcellular structural bases for row‐specific cytoarchitecture and innervation pattern in mouse cochlear amplifier.

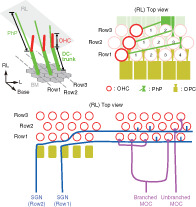

## 1 INTRODUCTION

In the mammalian cochlea, sound pressure changes are converted into traveling waves on the basilar membrane (BM). The resultant vibration is then transduced by the organ of Corti (OC), which is arranged along the coiled BM, into electrical signals in auditory nerve fibers (ANFs). Two types of hair cells reside in the OC, with inner hair cells (IHCs) being the true sensory cell type for detecting the BM motion and coding the acoustic information. In contrast, outer hair cells (OHCs), which are located on the lateral side of the OC, are responsible for modulating the BM motion via their electromotility and in turn the IHC sensation. This remarkable structure plays an essential role in the cochlear amplification for the perception of low‐level sounds (Dallos et al., 1996; Meyer & Moser, 2010; Raphael & Altschuler, 2003).

There are three rows of OHCs docked on a honeycomb‐like network of supporting Deiters cells (DCs) along the BM. At their epithelial surface, the reticular lamina (RL), OHCs and the phalangeal processes (PhPs) from the DCs are adjoined and precisely arranged in a mosaic‐like organization. As the PhPs are tilted in the opposite direction of the OHCs, it yields between the RL and BM a lattice of characteristic Y‐shaped structures arranged in the direction of the basilar membrane's traveling wave. The resultant structural coupling between the electromotile OHCs and passive PhPs, as proposed in prior studies (Motallebzadeh et al., 2018; Wen & Boahen, 2003; Yoon et al., 2011), has an important effect on the mechanical behavior of the cochlea. However, early structural investigations using electron microscopy (EM) were limited by either specimen volume or structure accessibility. By far, the most knowledge about this Y‐shaped building block was gained from the third (outermost) OHC‐DC row in fixed OC of mole rat (Raphael et al., 1991), hence lacking anatomical information of the first two rows (inner rows). More recent quantification in the mouse cochlea using in situ two‐photon imaging revealed significant differences in structural details of the Y‐shapes between rows, including longitudinal angles and PhP lengths (Soons et al., 2015). Assuming negligible changes in tissue morphology caused by opening the cochlea, this result indicates putative heterogeneous mechanical properties of the Y‐shapes in different rows.

Moreover, the OHC motility is tightly regulated by centrifugal innervations to enable signal discrimination in a noisy background and binaural signal localization (see reviews, Fuchs & Lauer, 2019; Wersinger & Fuchs, 2011; Zhang & Coate, 2017). At the OHC basal pole, DC forms a cup‐like structure housing both afferent and efferent projections of the central auditory nervous system. In mammals, thin unbranched afferent fibers of type‐2 spiral ganglion neurons (SGNs) turn 90° toward basal cochlear region after crossing the floor of the tunnel of Corti (TC) and travel a characteristic 100–200 μm distance in parallel to the OHC rows before making synaptic contact (Berglund & Ryugo, 1987; Dannhof & Bruns, 1993; Spoendlin, 1972). Currently, much less is known about the exact function of the type‐2 SGN and the linkage to its characteristic anatomical features. Several concepts have been proposed including auditory nociception (Flores et al., 2015), cochlear damage report (Liu et al., 2015) as well as efferent control (Froud et al., 2015; Maison et al., 2016). In addition, OHCs are also the primary targets of medial olivocochlear (MOC) efferent neurons (Warr et al., 1997). The MOC fibers cross at the middle level of the TC and synapse onto multiple OHCs with an unusual inhibitory action of acetylcholine (Blanchet et al., 1996; Dallos et al., 1997; Evans et al., 2000; Wersinger & Fuchs, 2011). In rat, two subtypes of MOC fibers were identified based on their abundance of the tunnel‐crossing fibers and boutons (Warr & Boche, 2003). However, it remains unclear the functional implication behind such diversity of axonal ramifications.

In order to elucidate the structural basis of the cochlear amplifier as well as its circuit wiring principle, we performed comprehensive ultrastructural analysis in large‐scale EM volume of mouse mid‐cochlea region using serial block‐face EM (SBEM, Denk & Horstmann, 2004). Our results including several structural insights are relevant for biophysical modeling of BM motion as well as understanding the cochlear amplifier modulation.

## 2 MATERIALS AND METHODS

### 2.1 Animals

CBA/Ca female mice (p49, p60) were purchased from Sino‐British SIPPR/BK Lab.Animal Ltd (Shanghai, China). This study was conducted at the Shanghai Institute of Precision Medicine and Ear Institute of Shanghai Ninth People's Hospital. All procedures were reviewed and approved by the Institutional Authority for Laboratory Animal Care of the hospital (SH9H‐2019‐A387‐1).

### 2.2 Sample preparation

SBEM sample preparation was performed following the previously published procedure (Hua et al., 2021). In brief, fresh temporal bones were harvested from adult CBA mice. The cochlea was fixed by perfusion with ice‐cold fixative mixture (2% paraformaldehyde and 2.5% glutaraldehyde buffered in 0.08 M cacodylate, pH 7.4) through round window followed by a 5‐hour postfixation at 4°C. Decalcification was done by 4‐hour immersion in the same mixture with addition of 5% EDTA. The decalcified cochleae were washed twice then *en bloc* EM stained by sequentially incubating in 0.15 M cacodylate buffer (pH 7.4) containing 2% OsO_4_, 2.5% ferrocyanide, and again 2% OsO_4_ at room temperature for 2, 2, and 1.5 hours, respectively. After two 30‐min washes with nanopore‐filtered water, the cochleae were incubated at room temperature in filtered thiocarbonhydrazide (saturated aqueous solution) for 1 hour, unbuffered OsO_4_ aqueous solution for 2 hours and lead aspartate solution (0.03 M, pH 5.0 adjusted by KOH) at 50°C for 2 hours with intermediate washing steps. Dehydration and embedding were done through a graded acetone series (50%, 75%, 90%, 30 min each, all cooled at 4°C) into pure acetone (three times 100%, 30 min at room temperature) followed by sequential infiltration with 1:1 and 1:2 mixtures of acetone and Spurr's resin monomer (4.1 g ERL 4221, 0.95 g DER 736, 5.9 g NSA, and 1% Dimethylaminoethanol) and the pure resin at room temperature for 6 hours each. The infiltrated cochleae were then placed in embedding molds and incubated in a prewarmed oven (70°C) for 72 hours.

### 2.3 Electron microscopy

Resin‐embedded cochleae were trimmed down to a final block‐face size of ~800 × 800 μm^2^ and mounted on an in‐chamber ultramicrotome (3ViewXP, Gatan). Imaging acquisition was done using a field‐emission scanning EM (Gemini300, Zeiss) with back‐scattered electron detector (Onpont, Gatan). For the data acquisition, serial images were registered in single‐tile mode (20,000 × 15,000 pixels) at 11 nm pixel size and a nominal cutting thickness of 40 nm; incident beam energy 2 keV; dwell time 1 μs. Focal charge compensation (Deerinck et al., 2018) was set to 100% with a vacuum chamber pressure of ∼ 2.8 × 10^−3^ mbar. In total, 5193 consecutive slices were collected and aligned using self‐written MATLAB script based on cross‐correlation maximum, yielding an EM volume of 325.0 × 204.4 × 207.7 μm^3^.

### 2.4 Cytoarchitecture reconstruction and data processing

Structure inspection and annotation were done in a browser‐based annotation tool, webKNOSSOS (Boergens et al., 2017). Each neurite was at least two‐fold traced by multiple observers. The results of the first annotator were proofread by another one for missing branches and for few confusing cases a third annotator was involved in voting. For quantification of geometrical features such as 3D angle and length, landmarks were created: OHCs were skeletonized along the RL‐BM axis; DCs and PhPs were annotated by cytoskeleton tracing; the plane of BM was determined by the roots of DCs; the intersection angle (α) between two branches of Y‐shapes were measured from vectorized OHC and PhP (*svd*, MATLAB build‐in function). Longitudinal angles of OHCs, PhPs, DC main trunks, and OPCs were measured in reference to the linear‐fitted DC rows. Efferent and afferent fibers were identified based on their characteristic morphology such as fiber trajectory and the presence of presynaptic bouton filled with vesicle cloud.

### 2.5 Statistics

**FIGURE 1:**
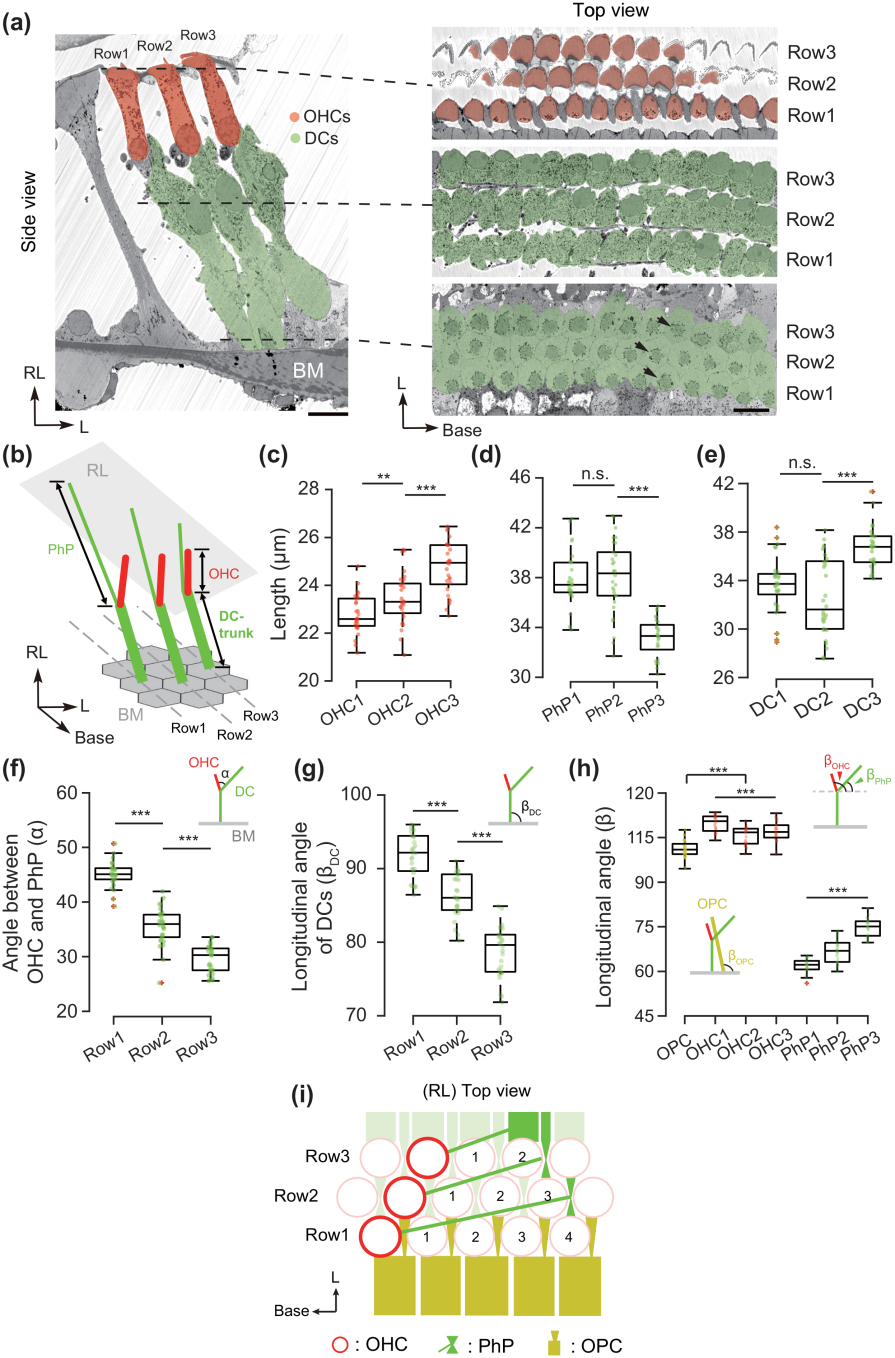
Structural heterogeneity of Y‐shapes. (a) Side view (left) of the cytoarchitecture of mouse cochlear amplifier with outer hair cells (OHCs) in red and Deiters cells (DCs) in green. In the top view (right) at different level, both OHCs and DCs are arranged in a honeycomb‐like pattern with microtubule bundles (arrows). Scale bar, 10 μm. (b) Schematic illustration of quantified structures including lengths of phalangeal process (PhP) (L_PhP_), OHC (L_OHC_), and DC main trunk (L_DC_). (c) The lengths of OHCs in different rows. OHC1: 22.80 ± 0.80 μm (*n* = 29), OHC2: 23.45 ± 1.05 μm (*n* = 30), OHC3: 24.75 ± 1.06 μm (*n* = 29). Two‐sample *t*‐test: **p = .0092 (OHC1 vs. OHC2); ***p < .001 (OHC2 vs. OHC3). One‐way ANOVA: ***p < .001. (d) The lengths of PhPs in different rows. PhP1: 37.86 ± 2.11 μm (n = 29); PhP2: 38.01 ± 2.90 μm (*n* = 30); PhP3: 33.18 ± 1.51 μm (*n* = 29). Two‐sample *t*‐test: *p* = .8170 (PhP1 vs. PhP2); ***p < .001 (PhP2 vs. PhP3). One‐way analysis of variance (ANOVA): ***p < .001. (e) The lengths of DC main trunks in different rows. DC1: 33.65 ± 2.28 μm (*n* = 29); DC2: 32.57 ± 3.18 μm (*n* = 30); DC3: 36.61 ± 1.68 μm (*n* = 29). Two‐sample *t*‐test: *p* = 0.1411 (DC1 vs. DC2); ***p < .001 (DC2 vs. DC3). One‐way ANOVA: ***p < .001. (f) The intersection angles between the OHC and PhP (α in inset). The first row: 45.58 ± 2.29° (*n* = 29); the second row: 36.09 ± 3.37° (*n* = 30); the third row: 30.22 ± 2.44° (*n* = 29). Two‐sample *t*‐test: ***p < .001 (Row1 vs. Row2); ***p < .001 (Row2 vs. Row3). One‐way ANOVA: ***p < .001. (g) The longitudinal angles of DC main trunks (β_DC_ in inset): DC1: 91.77° ± 3.01° (*n* = 28); DC2: 86.38 ± 2.98° (*n* = 28); DC3: 78.73 ± 3.45° (*n* = 28). Two‐sample *t*‐test: ***p < .001 (DC1 vs. DC 2); ***p < .001 (DC2 vs. DC3). One‐way ANOVA: ***p < .001. (h) The longitudinal angles of OPCs (β_OPC_), OHCs (β_OHC_) and PhPs (β_PhP_). OPC: 101.04° ± 3.04° (*n* = 32); OHC1: 109.79 ± 3.07° (*n* = 28); OHC2: 105.43 ± 2.92° (*n* = 28); OHC3: 107.23 ± 3.35° (*n* = 28); OHC1‐3: 107.28° ± 3.44° (*n* = 84): PhP1: 61.45° ± 2.48° (*n* = 28); PhP2: 66.58 ± 3.75° (*n* = 28); PhP3: 75.01 ± 3.25° (*n* = 28). Two‐sample *t*‐test: ***p < .001 (OPC vs. OHC1‐3). One‐way ANOVA: ***p < .001 (OHCs); ***p < .001 (PhPs). (i) Schematic illustration of the arrangement of OHCs and PhPs at the RL. The span over the OHC (red) and the PhP (green) of the same Y‐shape is four, three and two OHC columns for the first, second, and third row, respectively [Color figure can be viewed at wileyonlinelibrary.com]

**FIGURE 2:**
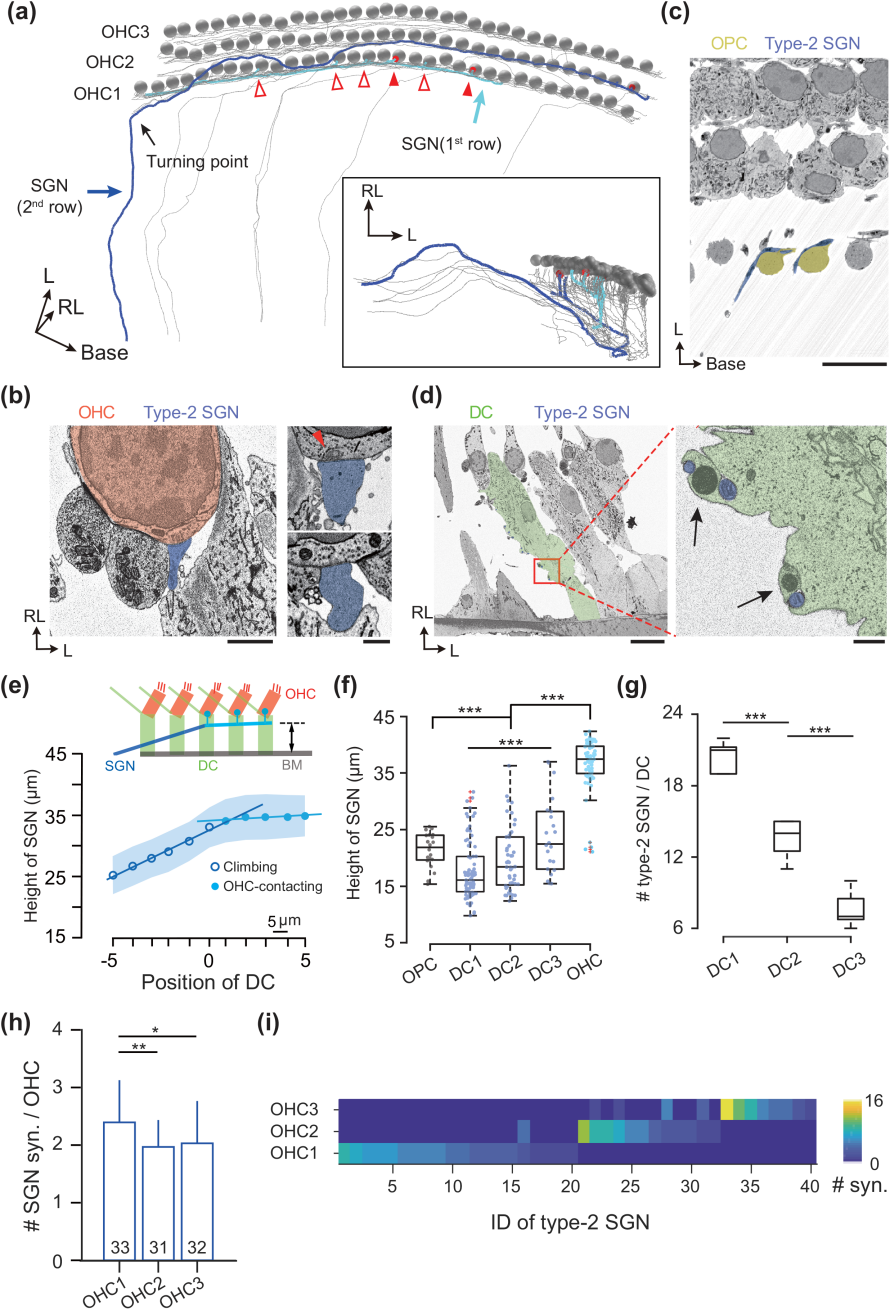
Fiber morphology and row‐specific innervation of type‐2 spiral ganglion neurons (SGNs). (a) Top view (left) and side view (inset) of the reconstructed type‐2 SGNs (blue) with characteristic turning toward the basal cochlear region (black arrow). They synapse with three outer hair cells (OHC) rows (gray spheres) via short collaterals. Two example SGNs which synapse exclusively with OHCs in the first (dark blue) and the second row (light blue) were illustrated. Ribbon‐associated (red dots, filled arrow heads) and ribbon‐less terminals (open arrow heads) were indicated. (b) Representative electron micrograph (left, side view) of a contact between type‐2 SGN (blue) and OHC (red). Scale bar, 2 μm. Both ribbon‐associated terminals (top right) and ribbon‐less terminals (bottom right) were observed. Scale bar, 1 μm. (c) Electron micrograph (top view) showing the turning of type‐2 SGN (blue) around outer pillar cells (OPCs) (yellow). Scale bar, 10 μm. (d) Electron micrograph (side view) showing Deiters cells (DCs) (green, the first row) with attached type‐2 SGN fibers (blue). Scale bar, 10 μm. Inset: Anchor‐like structures of the DC (green) for interacting with the SGN fibers (blue). Scale bar, 1 μm. Mitochondria were indicated by arrows. (e) Vertical height of individual SGN fiber on bypassed DCs (from the contact site to the basilar membrane [BM]). Linear‐fit of height changes in the SGN functional compartments resulted in two distinct slopes, that is for climbing region (circles) 0.23 ± 0.07 (1.56 ± 0.44 μm per DC, *n* = 15) as well as for OHC‐contact region (dots) 0.02 ± 0.09 (0.20 ± 0.51 μm per DC, *n* = 15). Paired *t*‐test: ***p < .001 for slopes; ***p < .001 for height changes. Scale bar for x‐axis 5 μm. (f) Height distribution of the SGN contact sites on OPCs and DCs. The collateral‐free climbing region (DC1‐DC3) and the OHC‐contact region (OHC) of SGNs were plotted separately. On OPC: 21.39 ± 3.06 μm (*n* = 20); on DC1: 17.81 ± 5.23 μm (*n* = 77); on DC2: 19.73 ± 5.64 μm (*n* = 48); on DC3: 23.85 ± 6.68 μm (*n* = 22); for the SGN (OHC‐contact region) on DCs: 36.53 ± 4.72 μm (*n* = 61). Two‐sample *t*‐test: ***p < .001(OPC vs. DC1‐3); ***p < .001 (DC1‐3 vs. syn.); ***p < .001 (DC1 vs. DC3); **p = .0094 (DC2 vs. DC3). One‐way analysis of varaince (ANOVA): ***p < .001 (DCs); ***p < .001 (OPC, DC1‐3, and OHC). (g) Abundance of type‐2 SGNs on DCs of different rows. DC1: 20.4 ± 1.3 (*n* = 5); DC2: 13.6 ± 1.7 (*n* = 5); DC3: 7.6 ± 1.5 (*n* = 5). Two‐sample *t*‐test: ***p < .001 (DC1 vs. DC2); ***p < .001 (DC2 vs. DC3). One‐way ANOVA: ***p < .001. (h) Mean SGN contacts on each OHC. OHC1: 2.39 ± 0.70 (*n* = 33); OHC2: 1.97 ± 0.48 (*n* = 31); OHC3: 2.03 ± 0.69 (*n* = 32). Two‐sample *t*‐test: **p = .0067 (OHC1 vs. OHC2) and *p = .0407 (OHC1 vs. OHC3). (i) Heat map showing row‐specific OHC‐innervation of SGNs. Only five out of 40 (12.5%) SGNs sample OHCs from two neighboring rows, while the remaining 35 SGNs (87.5%) exclusively contact OHCs within the same row [Color figure can be viewed at wileyonlinelibrary.com]

**FIGURE 3:**
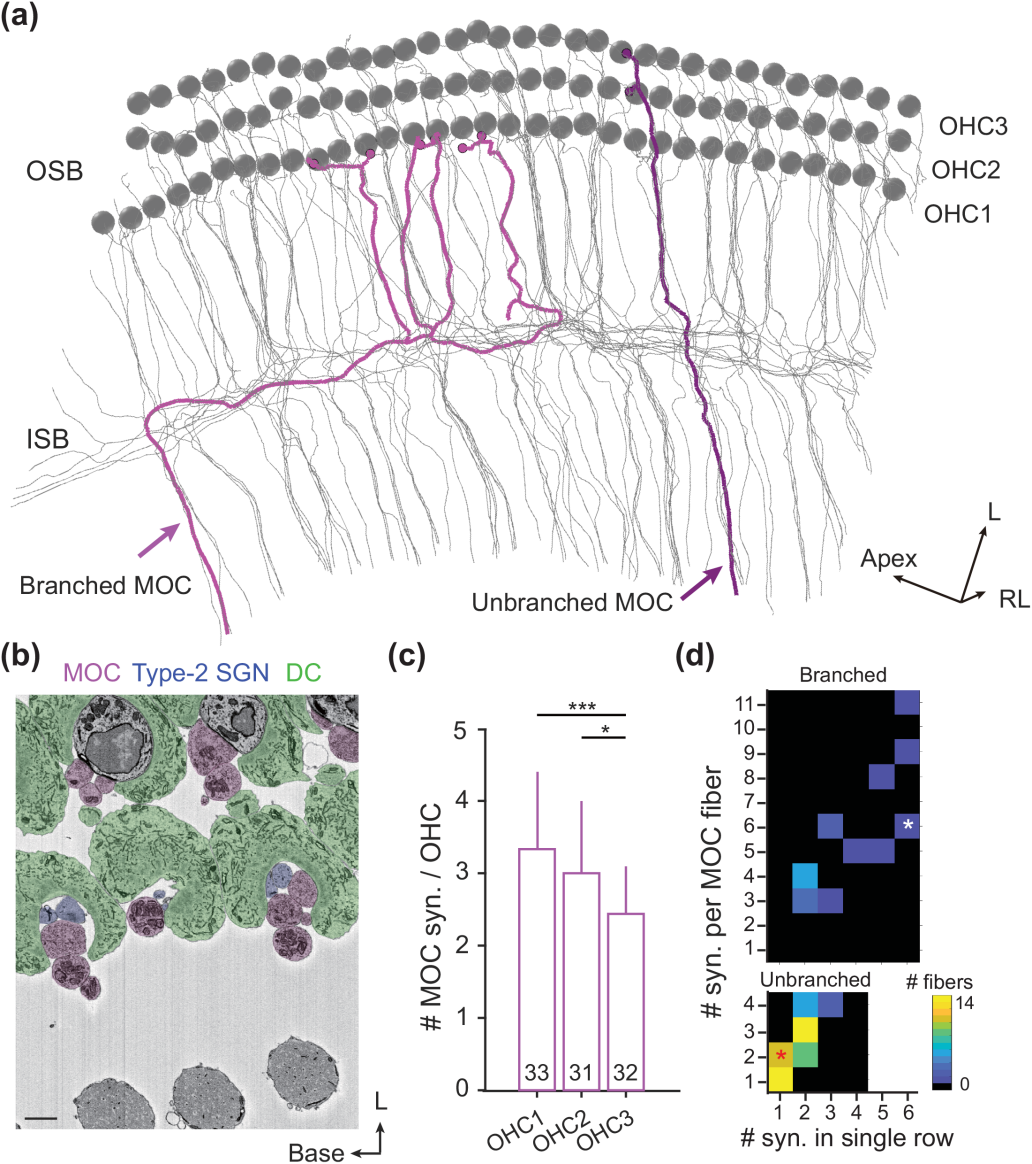
Innervation of efferent medial olivocochlear (MOC) fibers. (a) Top view of all 70 skeletonized MOC fibers (gray). Representative branched (light magenta) and unbranched fibers (dark magenta) with boutons (magenta dots) onto outer hair cells (OHCs) (gray spheres). (b) Electron micrograph (top view) showing Deiters cells (DC)‐cups (green) which accommodate the presynaptic boutons of MOCs (magenta) and the postsynaptic spiral ganglion neuron (SGN) terminals (blue). Scale bar, 1 μm. (c) Average MOC innervations per OHC. OHC1: 3.33 ± 1.05 (*n* = 33); OHC2: 3.00 ± 1.03 (*n* = 31); OHC3: 2.44 ± 0.67 (*n* = 32). Two‐sample *t*‐test: *p* = .205 (OHC1 vs. OHC2); ***p < .001 (OHC1 vs. OHC3); *p = .0125 (OHC2 vs. OHC3). (d) Total vs. row‐specific innervations on OHCs measured by the number of synapses in individual branched (top) and unbranched MOC fibers (bottom). Heat map represents the number of fibers sharing different degrees of row‐specificity. Two example fibers shown in (a) were indicated by asterisks (white for the branched and red for the branched fiber) [Color figure can be viewed at wileyonlinelibrary.com]

All data analysis including statistical tests were performed using self‐written script in MATLAB including built‐in function and the Statistics Toolbox (MathWorks, Inc.). The group comparisons were done using two‐sample *t*‐test (*ttest2*) for Figures 1(c–h), 2(f–h), and 3(c), one‐way analysis of variance (ANOVA) (anova1) for Figures 1(c–h), 2(f–h), and 3(c), as well as paired *t*‐test (*ttest*) for Figure 2(e). Data were reported as mean ± SD and the significance level of statistical tests was denoted as n.s. for *p*‐value > .05, * for *p* < .05, ** for *p* < .01, and *** for *p* < .001.

## 3 RESULTS

The SBEM volume with a size of 325.0 × 204.4 × 207.7 μm^3^ at 11 × 11 × 40 nm^3^ pixel size was acquired from mid‐cochlea region of a 7‐week‐old CBA mouse. The outer spiral bundle (OSB) region contains 96 OHCs, 96 DCs, 32 outer pillar cells (OPCs), 40 type‐2 SGNs as well as 70 MOC fibers.

### 3.1 Cytoarchitecture of the Y‐shaped OHC‐DC complex

The large EM volume allowed quantitative structural analysis of the 88 intact OHC‐DC complexes in 3D (Figure 1(a)) with 29 in the first row (innermost), 30 in the second row (middle), and 29 in the third row (outermost). We skeletonized the OHCs, OPCs and the characteristic microtubule bundles in the DCs as shown in Figure 1(b). Slightly increased OHC length was found across three rows with the longest OHC in the third row (Figure 1(c), OHC1: 22.80 ± 0.80 μm; OHC2: 23.45 ± 1.05 μm; OHC3: 24.75 ± 1.06 μm), consistent with previous OHC length measurement (Dannhof et al., 1991) in the middle turn region (50%–60% of the cochlear length, frequency range of 12–16 kHz). For the PhPs, similar length was observed from the first two rows, while DCs in the third row have significantly shorter PhPs (Figure 1(d), PhP1: 37.86 ± 2.11 μm; PhP2: 38.01 ± 2.90 μm; PhP3: 33.18 ± 1.51 μm). However, this inter‐row difference is partially compensated by the DC main trunks (Figure 1(e), DC1: 33.65 ± 2.28 μm; DC2: 32.57 ± 3.18 μm; DC3: 36.61 ± 1.68 μm). Next, the longitudinal tilt of the OHCs, PhPs and DCs was measured. From the first to the third row, the PhPs become more perpendicular to the BM (PhP1: 61.45° ± 2.48°; PhP2: 66.58° ± 3.75°; PhP3: 75.01° ± 3.25°, Figure 1(h)), while the tilt of OHCs is similar between rows (OHC1‐3: 107.28° ± 3.44°, Figure 1(h)). This leads to a gradually reduced intersection angle (α, Figure 1(f) inset) between OHC and PhP from the first to the third row (Figure 1(f), Row1: 45.58° ± 2.29°; Row2: 36.09° ± 3.37°; Row3: 30.22° ± 2.44°). In contrast to a fixed longitudinal angle (β_DC_, Figure 1(g) inset) of about 120° between the BM and the DCs, reduced longitudinal tilt was observed with largest angle in the first row (Figure 1(g), DC1: 91.77° ± 3.01°; DC2: 86.38° ± 2.98°; DC3: 78.73° ± 3.45°). Interestingly, OPCs were found flanked toward the basal cochlear region but slightly more perpendicular to the BM than the OHCs (Figure 1(h), OPC: 101.04 ± 3.04°, *n* = 32), providing presumably additional pushing and pulling forces.

These results described structural heterogeneity of OHC‐DC complexes in different rows. Considering the hexagonal honeycomb‐like arrangement at the base of DCs, how are the Y‐shapes accommodated at the RL? We found regularly the PhPs in the first row (29/29 OHC‐DC complexes) span over four OHC1s, abutting with one OHC1, two OHC2s and one OHC3; the PhPs in the second row (29/30 OHC‐DC complexes) span over three OHCs, abutting with one OHC2 and two OHC3s; the PhPs in the third row (29/29 OHC‐DC complexes) span over only two OHC3, next to one OHC3 (Figure 1(i)). This fine cytoarchitecture constrains the intersection angle (α, Figure 1(f) inset) between OHC and PhP of the Y‐shapes in different rows.

### 3.2 Morphology and innervation of type‐2 SGNs

Together with previous studies, our structural data have quantified the inter‐row difference in OHC‐DC complexes in mouse. It remains to elucidate whether the nerve circuit of cochlear amplifier is tuned in a row‐specific fashion. In our EM volume, morphology and innervation pattern of type‐2 SGNs were studied by dense fiber reconstruction (Figure 2(a)) and bouton annotation (Figure 2(b)). In line with prior report in rat (Martinez‐Monedero et al., 2016), the percentage of ribbon‐associated boutons in our mouse dataset (Figure 2(b) inset) was 31.6% (*n* = 33), 31.1% (*n* = 31) and 53.8% (*n* = 32) from the first to the third OHC row. The type‐2 SGNs feature a sharp turning of 90° right after bypassing the OPCs (Figure 2(c)) and then travel next to DCs with an anchor‐like structure (Figure 2(d) and Supplementary video S1). Based on the presence of OHC‐innervating collaterals, the type‐2 fibers were divided into two functional compartments. Individual fiber trajectories show an increasing monotonic relationship with a steep slope in bouton‐free regions and ultimately plateauing when forming afferent contacts onto OHCs (Figure 2(e), 0.23 ± 0.07 for bouton‐free and 0.02 ± 0.09 for bouton‐rich trajectory, *n* = 15). This data implies that the raising trajectory of type‐2 SGN fibers may be structurally regulated by DCs. When pooling the height of type‐2 SGNs from BM on randomly selected OPCs and DCs, we found a gradual upshift of SGN distribution on DCs from the first to the third row after initial drop from OPCs. Contacts between OHCs and SGN collaterals occurs only when SGN main trunk reaches certain height on DCs (Figure 2(f), on OPC: 21.39 ± 3.06 μm, *n* = 20; on DC1: 17.81 ± 5.23 μm, *n* = 77; on DC2: 19.73 ± 5.64 μm, *n* = 48; on DC3: 23.85 ± 6.68 μm, *n* = 22; OHC‐contacted: 36.53 ± 4.72 μm, *n* = 61). Given that the relative abundance of afferent fibers per DC decreases from DC1 to DC3 (Figure 2(g), DC1: 20.4 ± 1.3, *n* = 5; DC2: 13.6 ± 1.7, *n* = 5; DC3: 7.6 ± 1.5, *n* = 5), our result suggests an out‐spiral morphology of type‐2 SGNs across DC rows. We next focused on the OHC innervation of SGNs and found that on average the first row OHCs contact with 20% more afferent terminals than those in the second and third rows (Figure 2(h), OHC1: 2.39 ± 0.70, *n* = 33; OHC2: 1.97 ± 0.48, *n* = 31; OHC3: 2.03 ± 0.69, *n* = 32). Moreover, type‐2 SGNs sample predominantly from OHCs of the same row (Figure 2(i)).

### 3.3 Innervation of MOC fibers on OHCs

Unlike type‐2 SGNs, which showed a height differences with respect to synapse locations, efferent MOC fibers traverse the TC radially (in largely the same plane) contacting the basal pole of the OHCs where they form synaptic contacts (Figure 3(a)). As previously described, nerve endings were physically supported by a cup‐like structure where the basal pole of OHC is docked (Figure 3(b)). A radial gradient of decreasing efferent contacts per OHC was found from the first to the third row (Figure 3(c), OHC1: 3.33 ± 1.05, *n* = 33; OHC2: 3.00 ± 1.03, *n* = 31; OHC3: 2.44 ± 0.67, *n* = 32). Further analysis classified MOC neurons into branched and nonbranched subtypes based on the abundance of tunnel‐crossing fibers (see Figure 3(a) for examples). In the mouse mid‐cochlea region, the majority of MOC neurons (75.7%, 53/70) features a single tunnel‐crossing fiber with an output synapse number ranging from 1 to 4 (2.26 ± 1.00), while 24.3% (17/70) were classified as the branched subtype with significantly more output synapses (5.18 ± 2.30, range from 3 to 11). In good agreement to prior study in mice (Wilson et al., 1991), these MOC fibers form a total of 215 nerve endings on OHCs of different rows (first row: 37.0%, *n* = 77 synapses; second row: 35.6%, *n* = 74; third row: 27.4%, *n* = 57). Next, we analyzed the innervation specificity of individual fiber. In contrast to the unbranched MOC fibers of equal innervation probability on three OHC rows (OHC1: 34.4%, *n* = 41 synapses; OHC2: 35.3%, *n* = 42; OHC3: 30.3%, *n* = 36), the branched fibers, in spite of large inter‐fiber variation, synapse preferentially with OHCs of the first two rows (OHC1: 40.0%, *n* = 36; OHC2: 36.7%, *n* = 33; OHC3: 23.3%, *n* = 21). Interestingly, branched MOC fibers, but not unbranched fibers, distribute the majority of their synapses in a single OHC row (Figure 3(d)), indicating a strong row‐specific innervation.

### 3.4 Confirmation of structural findings in second animal

**FIGURE 4:**
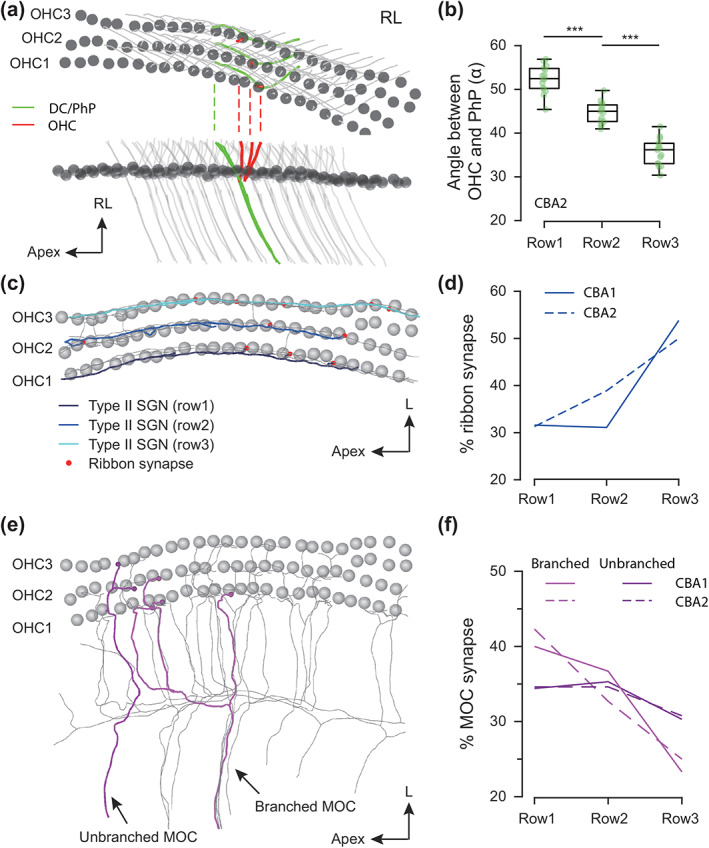
Comparison with the second CBA animal. (a) Top view (top) and side view (below) of skeletonized outer hair cells (OHCs) and Deiters cells (DCs). One example column of OHCs (red) and DCs (with PhP, green) were highlighted in colors. Note different intersection angles between OHC and PhP in different rows. (b) The intersection angles between the OHC and PhP in the second CBA animal. The first row: 52.36 ± 3.14° (*n* = 15); the second row: 44.73 ± 2.45° (*n* = 15); the third row: 35.65 ± 3.07° (*n* = 15). Two‐sample *t*‐test: ***p < .001 (Row1 vs. Row2); ***p < .001 (Row2 vs. Row3). One‐way ANOVA: ***p < .001. (c) Top view of the traced type‐2 SGNs in the second CBA (gray). Three representative fibers (Row1: Dark blue; Row2: Blue; Row3: Light blue) were illustrated with row‐specific innervation and ribbon‐associated terminals (red). (d) Comparison of the percentage of ribbon‐associated terminals between the first CBA (solid line, Row1: 31.6%, *n* = 33; Row2: 31.1%, *n* = 31; Row3: 53.8%, *n* = 32) and the second CBA animal (dashed line, Row1: 31.3%, *n* = 16; Row2: 38.9%, *n* = 18; Row3: 50.0%, *n* = 24). (e) Side view of the traced MOC‐fibers (gray) with examples of branched MOC (magenta) and unbranched MOC (violet). (f) Interspecimen comparison of the percentage of MOC synapses onto different rows. For the branched MOCs in CBA1 (magenta solid line): 40.0% (Row1, *n* = 36), 36.7% (Row2, *n* = 33) and 23.3% (Row3, *n* = 21) and in CBA2 (magenta dashed line): 42.3% (Row1, *n* = 22), 32.7% (Row2, *n* = 17) and 25.0% (Row3, *n* = 13). For the unbranched MOCs in CBA1 (violet solid line): 34.4% (Row1, *n* = 41), 35.3 (Row2, *n* = 42) and 30.3% (Row3, *n* = 36) and in CBA2 (violet dashed line): 34.6% (Row1, *n* = 9), 34.6% (Row2, *n* = 9) and 30.8% (Row3, *n* = 8) [Color figure can be viewed at wileyonlinelibrary.com]

In order to validate these structural findings, we analyzed randomly sampled structures in a SBEM dataset acquired from another CBA animal (p60, middle turn). The volume is 214.8 × 252.6 × 147.6 μm^3^ sized at slight lower resolution (12 × 12 × 50 nm^3^). In total 45 Y‐shapes (Figure 4(a)), 10 type‐2 SGNs (Figure 4(c)) as well as 16 MOC‐fibers (Figure 4(e)) were annotated. Note that a small region with four rows of OHCs was excluded in the analyses. First, we observed comparable structural changes in Y‐shapes across OHC rows (Table 1) and the identical cellular organization (Figure 1(i)) at the RL from the second animal (CBA2) which leads to the same trend in the intersection angles between OHCs and PhPs with the largest values in the innermost row (Figure 4(b), Row1: 52.36° ± 3.14°; Row2: 44.73° ± 2.45°; Row3: 35.65° ± 3.07°). Second, row‐specific innervation pattern of type‐2 SGNs was consistent for both animal with similar fraction of ribbon‐associated terminals (Figure 4(d), for CBA2, Row1: 31.3%, *n* = 16 boutons; Row2: 38.9%, *n* = 18; Row3: 50.0%, n = 24). Third, similar to the first animal (CBA1), the branched MOC‐fibers rather than the unbranched fibers innervate preferentially the first two OHC rows in CBA2 (Figure 4(f), branched fibers onto OHC1‐3: 42.3%, 32.7%, and 25.0% versus unbranched fibers: 34.6%, 34.6%, and 30.8%.

**TABLE 1:** Comparison of Y‐shapes between two mice

	CBA1				CBA2			
	Row1	Row2	Row3	ANOVA test 1	Row1	Row2	Row3	ANOVA test 1
L_OHC_ (μm)	22.80 ± 0.80	23.45 ± 1.05	24.75 ± 1.06	***p < .001	23.26 ± 0.72	22.79 ± 0.92	23.26 ± 0.83	*p* = .2154
L_PhP_ (μm)	37.86 ± 2.11	38.01 ± 2.90	33.18 ± 1.51	***p < .001	36.94 ± 1.58	33.96 ± 1.67	30.84 ± 1.58	***p < .001
L_DC_ (μm)	33.65 ± 2.28	32.57 ± 3.18	36.61 ± 1.68	***p < .001	38.09 ± 1.78	39.72 ± 1.41	42.31 ± 1.40	***p < .001
α_OHC‐PhP_ (°)	45.58 ± 2.29	36.09 ± 3.37	30.22 ± 2.44	***p < .001	52.36 ± 3.14	44.73 ± 2.45	35.65 ± 3.07	***p < .001
β_DC_ (°)	91.77 ± 3.01	86.38 ± 2.98	78.73 ± 3.45	***p < .001	86.98 ± 1.93	81.02 ± 2.03	73.64 ± 1.71	***p < .001

*Note*: Data are presented as mean ± SD.

The significance level of statistical tests was denoted as *** for *p* < .001

## 4 DISCUSSION

Serial section EM approaches have powered 3D ultrastructure investigations in both inner and outer hair cells (Anttonen et al., 2014; Bullen et al., 2015; Dannhof & Bruns, 1993; Fuchs et al., 2014; Glueckert et al., 2005; Hashimoto et al., 1990; Hua et al., 2021; Liberman et al., 1990; Thiers et al., 2008). In this study, we presented large‐scale cytoarchitecture of the mouse cochlear amplifier revealed by SBEM technique (Denk & Horstmann, 2004). To the best of our knowledge, the ultrastructural quantifications in the OSB region were for the first time achieved for a BM of more than 200 μm in length. Extended structural insights were gained in well‐organized DC framework, type‐2 SGN morphology as well as row‐specific tuning of afferents and efferents on OHCs, allowing model refinement of the mouse OC in 3D. In comparison to dissecting and flattening cochlear tissues, whole cochlea *en bloc* preparation has the advantages in preserve 3D structural information and minimize mechanical damage. However, it is known that slow chemical fixation can introduce artifacts mainly tissue shrinking. In our hands, best ultrastructure preservation was achieved by cochlear perfusion with ice‐cold fixative, which was further supported by largely comparable measurements to those obtained in vivo (Soons et al., 2015). Moreover, improved spatial resolution of EM compared to light microscopy allows visualization of structural features in cellular organization, fiber morphology as well as synaptic contact, enabling comparative structural investigations at neural circuit level in disease animal models of hearing loss, for example, *cochlear* synaptopathy (Kujawa & Liberman, 2009; Stamataki et al., 2006), cell death (Anttonen et al., 2014; Stamataki et al., 2006) and aging (Lauer et al., 2012; Sergeyenko et al., 2013).

In line with prior study (Soons et al., 2015), inter‐row structural differences in Y‐shaped OHC‐DC complexes were observed, including slightly longer OHC but significantly shorter PhP in the third row (Figure 1(c,d)). This is interesting, because it was found recently that DCs in the third row are transcriptionally different than those in the first two rows (Kolla et al., 2020). Unexpectedly, we found the RL span between OHC and PhP of the same DC is exact four, three and two OHC columns for the first, second, and third rows, respectively (Figure 1(i)), introducing distinct angles of the Y‐shapes in different rows, at least at the mid‐cochlear region. Our data strengthen the observation from previous study using two‐photon light microscopy (Soons et al., 2015) and argue for potential biological relevance behind the inter‐row heterogeneity, as such well‐organized cytoarchitecture is unlikely caused by tissue damage or deformation during opening the cochlea. Although coordinated movement of three rows of OHCs is believed essential for cochlear amplification, minimal contribution of the third row to the cochlear sensitivity was argued (Chen et al., 2008). Indeed, the observation of steeper upper branches of the third row Y‐shapes implies increased structural rigidity. In contrast, wide angle in the first two rows may allow more dramatic structure deformation upon increased shear force as calculated from the modeling (Liu et al., 2011). This feature may provide the reason why the first row OHCs are most vulnerable to permanent acoustic injury (Liberman & Dodds, 1984; Liberman & Kiang, 1978; Robertson, 1982; Robertson et al., 1980; Robertson & Johnstone, 1980; Yoshida & Liberman, 1999). Moreover, the OPCs, which are tilted in parallel to OHCs, form a crisscross pattern with DCs (Figure 1(e); Dallos et al., 1996) and thereby can provide extra support for the first row.

Owing to unprecedent large EM volume, comprehensive fiber anatomy of the mouse cochlear amplifier was accessible. Several interesting features in fiber morphology were found in the OSB. First, turning of the type‐2 SGN fibers towards the cochlear base occurs around the OPCs (Figure 2(c)) instead of, as generally thought, after entering the OSB region. This may suggest that OPC is the potential on‐site where type‐2 fiber receive its guidance cue for turning. Second, the observed biphasic trajectory of the fibers along DCs indicates two distinct functional compartments, namely the climbing and the synapsing region (Figure 2(e)). The anchor‐like structures on DCs (Figure 2(d)) not only secure surface attachment of the type‐2 fibers but also seem to position them in a well‐organized manner, distributing fibers with partially overlapped synapsing regions underneath their presynaptic partners, the OHCs. Considering almost row‐specific innervation of the type‐2 fibers (Figure 2(i)), inter‐row difference in the fraction of overlapped fibers is most likely the structural basis for heterogeneous afferents on OHCs (Figure 2(h)). If that is the case, an inter‐DC coordination is expected for regulated fiber positioning, presumably involving DC gap‐junction and OHC‐activity‐dependent cue during development.

Similar to afferents, more efferent terminals were found on OHCs of the first two rows (Figure 3(c)). This result, consistent with prior studies in guinea pig (Altschuler et al., 1984; Hashimoto & Kimura, 1988) and cat (Liberman et al., 1990; Thiers et al., 2008), implies a conserved radial gradient of centrifugal modulation of OHC motility across mammalian cochleae, which decreases from the first to the third OHC row. Further analysis based on classified MOC fibers revealed that this innervation bias was created by a minor population with multiple tunnel‐crossing fibers and strong row‐specific innervation (Figure 3(d)). As highly branched MOC fibers were found originate exclusively from ipsilateral brainstem in rat (Warr & Boche, 2003), our data suggest contra‐ and ipsilateral MOC fibers differ in OHC innervation pattern and thereby may modulate BM motion in different ways. However, it remains to elucidate the exact role of this feature for binaural loudness balancing (Froud et al., 2015) in complementing direct gain control of type‐1 SGNs by the lateral olivocochlear efferent fibers (Darrow et al., 2006; Maison et al., 2013).

In conclusion, large‐scale 3D EM reconstruction of mouse cochlear amplifier is presented in this study. It provides structural insights for the inter‐row heterogeneity in morphology of OHC‐DC complex as well as gradient innervation of the type‐2 SGNs and the MOC fibers, although possible implications of these structural observations remain to be determined by future studies.

## CONFLICT OF INTERESTS

The authors declare that they have no competing interests.

## AUTHORS CONTRIBUTION

Yunfeng Hua designed the study. Yunfeng Hua and Hao Wu supervised the study. Haoyu Wang, Shengxiong Wang, and Yan Lu conducted the study with the help of Miaoxin Qiu, Wenqing Huang, and Ying Chen in the data analysis. Yunfeng Hua wrote the manuscript with the help of Haoyu Wang, Shengxiong Wang, and Yan Lu. Other authors commented on the manuscript.

### 1 PEER REVIEW

The peer review history for this article is available at https://publons.com/publon/10.1002/cne.25137.

## Supporting information


**Appendix** **S1:** Supplementary Information

## Data Availability

Data Availability Statement: One large EM volume (CBA1) supporting this study is publicly available at https://wklink.org/1233. The dataset of second animal (CBA2) is available from the corresponding author (Y.H.) on request.
